# Flexible and Reconfigurable OFDM Implementation in DSP Platform for Various Purposes and Applications

**DOI:** 10.3390/s24092732

**Published:** 2024-04-25

**Authors:** Spyridon K. Chronopoulos

**Affiliations:** Electronics-Telecommunications and Applications Laboratory, Physics Department, University of Ioannina, 45110 Ioannina, Greece; spychro@gmail.com

**Keywords:** orthogonal frequency division multiplexing, OFDM, digital signal processor, DSP, civil, military, application, unmanned, UAV

## Abstract

In the modern technological era of sophisticated applications and high-quality communications, a platform of clever strategy and quickly updated systems is needed. It should be capable of withstanding the fastest emerging problems like signal attenuation and hostile actions intended to harm the whole network. The main contributions of this work are the production of an OFDM system (with low cost) that can sustain high-speed communications and be easily adjusted with new integrated code while exhibiting the feasibility of implementing a transmitter–receiver system in the same DSP and demonstrating the holistic approach with the qualitative integration of such an architecture in a warfare scenario. Specifically, in this research, the point of view is toward three facts. The first is to show a method of quick self-checking the operational status of a digital signal processor (DSP) platform and then the pedagogical issues of how to fast check and implement an updated code inside DSPs through simple schematics. The second point is to present the prototype system that can easily be programmed using a graphical user interface (GUI) and can change its properties (such as the transmitted modulated sinusoids—orthogonal frequency division multiplexing subcarriers). Alongside the presentation, the measurements are presented and discussed. These were acquired with the use of an oscilloscope and spectrum analyzer. The third point is to qualitatively show the application of such a system inside a modern warfare environment and to recommend various potential system responses according to the development of such a platform of reconfigurable implemented OFDM systems. The implementation was performed for two types of systems: (1) transmitter and (2) transmitter–receiver system. Notably, the system acts quickly with a delay of about 1 msec in the case of transmitting and receiving in the same DSP, suggesting excellent future results under real conditions.

## 1. Introduction

Digital signal processors (DSPs) are similar in many cases to microcontrollers since they include the integration of non-volatile and volatile memory and also peripheral connections with decreased dimensions (small footprints). These make DSPs ideal for embedded applications with the additional advantages of low power consumption requirements and ease of integration into mobile devices, thus exhibiting excellent performance in various applications. Specifically, their wide range of applications includes mobile phones, modems, fax, speech processing, and others. DSPs can be reprogrammable to have alternative functions for different applications on the same platform. Various procedures such as signal mixing, phase shifting, modulation, and others are designed at the software level and then embedded as code in DSPs. Also, the operating accuracy of the DSP system is much higher than that of an analog system. Also, the cost remains low since only developing the needed software is required. Usually, there is no need to build new hardware. The operation runs in real time. It means that the DSP processes various information following some event corresponding to data originating from its analog input. In contrast, non-real-time processing is not as demanding. In general, DSPs are not as easily affected by environmental changes (e.g., temperature change) as analog systems [1,2,3].

A representative family of DSPs used in this research is TMS320. This DSP family, particularly the TMS320C6x (where C6x corresponds to a model of the C6000 series), stands out for its speed and notable architecture. In turn, it leads to outstanding operations concerning the performance of demanding numerical calculations. The C6x architecture based on the very long instruction word (VLIW) “velociTI” makes this family very powerful in performance. Eight instructions can be recalled from memory in each cycle. For example, for a clock rate of 1 GHz, the C6416 can recall up to eight instructions of 32 bits in 1 ns [3].

Basic DSP systems can contain analog-to-digital converters (ADCs). ADCs convert the voltage of an incoming signal into a binary number (quantization). At the same time, during sampling, the rate is the number of conversions of analog voltage to a binary number per second. In particular, at the input of the ADC, there is usually a sample and hold circuit. The system sustains the signal voltage for a sampling period while performing the analog-to-digital conversion. These converters can be 8-bit (e.g., in telecommunications) or 16-bit (e.g., audio applications). After the ADC, the digital signal reaches the DSP (e.g., C6x). After the signal processing, there is an output via a digital-to-analog converter (DAC) usually located on the same electronic board. Each DAC has a characteristic input–output response. The response sets the output voltage for a corresponding binary input number. However, the produced output is stepwise, creating frequency components greater than the sampling frequency. Therefore, various included filters in DSP platforms act as basic stages. These are an antialiasing input filter to filter out erroneous signals (due to incorrect or non-observance of the Nyquist theorem) and an output filter to correctly reconstruct the processed signal. An example of sampling is the 44.1 KHz frequency used in audio CDs. This frequency means that each sample is acquired at an interval approximately equal to 0.023 msec (1/44.1), while that of 8 KHz means that the sample is stored every 0.125 (1/8) msec. It is apparent in the first case that the resulting signal consists of at least five times more samples (0.125/0.023) compared to the second [2,4].

This work reports general information about the used DSP system as well as the technical characteristics of the utilized platform (DSP development board). The paper presents the implementation of the proposed system made with a DSP platform. The DSP was operated either as an OFDM transmitter or transceiver. The implemented system included cyclic prefix (CP), zero padding (ZP), convolutional coding (transmitter), and a Viterbi decoder (receiver), and it produced information subcarriers which were measured in the frequency domain with a (real-time) spectrum analyzer. An oscilloscope estimated the total delay during the transmission–reception process for certifying the correct operation of the implemented system. Then, the oscilloscope was reused to compare a known signal (internal pulse generator) with the final (decoded) one.

The implemented system incorporated OFDM (multiplexing technique) that can be integrated into various systems as long as it is modified to meet the requirements of each operating standard. The construction of the OFDM system using a DSP embedding technique provides maximum flexibility to the telecommunications system designer. The flexibility lies in the fact that the designer can quickly solve problems created by possibly poor design, operating conditions, or even inadequate channel estimation. Therefore, there is no need to redesign the hardware or rebuild it. The quick fix is followed in these cases by rapid code integration into the desired digital signal processor (DSP) as long as proper debugging has already been performed. So, the system design is transferred shortly in a coded form to the development board, and then the system restarts free of its original operational problems. This process is possible since the systems containing this kind of hardware (DSP) are reprogrammable and can produce any form of signal in a frequency range.

Furthermore, this research study shows some potential applications for military usage and proposals of how an embedded system could be relevant to considerable electronic warfare issues. The flexibility factor of this system could be used to change the transmitted subcarriers on-air in a matter of a few seconds (and that is the duration of transferring the new code inside the prototype DSP platform). Also, with a future set of up-converters, filters, and oscillators, the orthogonal frequencies can be coded (e.g., turbo coding) and thus encrypted, providing a superior electronic environment of antiwarfare. On the other hand, it can also contribute to an apparatus of intense warfare against hostile systems by blinding and disabling them from locking the aerial, terrestrial, or even underwater platform carrying the proposed system. Consequently, the main contributions of this research are multiple and intended toward a holistic approach. They include:

- A prototype OFDM platform capable of producing and handling a vast number of subcarriers whose number can be limited only by the technical specifications of the utilized ADC and DAC.
- The OFDM platform uses double protection including zero padding (ZP) and cyclic prefix (CP).
- Low-cost production and maintenance of an exhibited DSP system that modifies its functions only with the use of code and can consequently be upgraded easily for every scenario.
- The speed of adaptation (for example, adjusting the produced OFDM subcarriers and even the whole system) can take up to several seconds in the worst-case scenario. In this operational frame where speed is vital, the overall system delay was found equal to 1 ms using a new technique (to the author’s knowledge) based on sending and receiving predetermined pulses.
- The integration of a whole OFDM transmitter–receiver system in the same DSP, thus measured on the LEDs’ pins, as the DAC (output) and ADC (input) were already in use by the transmitter–receiver system. It constitutes a type of emulation showing the method of measuring without the use of an ADC and DAC.
- Also, to the best of the author’s knowledge, this is the first time that the presentation of such a prototype (on a DSP) includes a viable warfare scenario based on the proposed concepts of implementation.

This paper has seven sections. Section 2 presents general DSP information, including the proposed integrated orthogonal frequency division multiplexing (OFDM) system. In Section 3.1, examples of the integration of simple schematics into the DSP are given, as well as the measurements that were carried out with the help of an oscilloscope. This section also serves pedagogically for properly showing the implementation technique and how to make the necessary inspections from a measurement aspect. Section 3.2 analyzes the procedure of integrating the OFDM system into a DSP. At the same time, the acquired measurements are shown by using a real-time spectrum analyzer (to visualize the carriers in the frequency domain) and an oscilloscope (time domain). Then follows a proposal of probable integration within a modern warfare environment. Afterward, a comparative study follows and then some restrictions are mentioned. Finally, the paper presents various conclusions and future goals.

## 2. General DSP Information—TMS320C6416Τ DSP Starter Kit (DSK)

This study used the TMS320C6416T DSP Starter Kit (DSK) [5,6] in the prototyping phase of the research as the OFDM system integration platform. It is low-cost and developed by Texas Instruments and Spectrum Digital. The DSK communicates with a computer via a USB port. The USB port programs the platform’s DSP. The TMS320C6416T DSK tools include Texas Instruments simulators and access to the Analysis Toolkit comprising cache analysis and a multi-event profiler. In general, through cache analysis, the use of this memory is optimized and, by extension, the performance of the respective application. Also, there are various tools to confirm the correct operation of the platform. The hardware multiply–accumulate (MAC) function, the capability of circular and inverted-bit addressing, as well as the Harvard architecture are parts of the DSP architecture and instruction set. All of the above are optimized for the best performance. The DSK contains the DSP, which can perform 8000 million instructions per second in applications designed for images, videos, networking, and more. Also, an expansion card slot and two 80-pin connectors dedicated to external peripherals and memory interfaces are included. In more detail, the features of the platform are mentioned below [3,7,8]:

- DSK size: 210 × 115 mm;
- Digital processor with operating frequencies up to 1 GHz;
- 1 MB internal RAM and thus 512 KByte Flash with an 8-bit interface;
- 16 MB SDRAM with a 64-bit interface; 4 programmable LEDS and four DIP switches;
- Expansion socket for plug-in modules;
- Onboard IEEE JTAG interface and built-in JTAG support over USB;
- Four 3.5 mm audio sockets are intended for a microphone, line input, output (line in, line out), and speaker;
- TLV320AIC23 16-bit stereo encoder circuit (analog interface circuit—AIC—codec) for analog input and output. The AIC uses sigma-delta technology which provides the ADC and DAC functions. This technology uses a 12 MHz system clock and a sampling rate with a range of 8–96 KHz;
- Typical output frequencies from 0 to 96 KHz (depending on the type of ADC);
- Collaboration with Code Composer Studio (CCS), which is an integrated development environment (IDE) for digital signal processing applications and supports real-time debugging. It also has graphical capabilities. Furthermore, software code (could be a telecommunication system) written in C or Assembly language, or partially developed from graphical programming (e.g., Simulink), can be integrated into the DSK;
- Single +5 V supply and onboard voltage regulators of 1.26 V for the DSP and 3.3 V for the memory and its peripherals.

**Figure 1:**
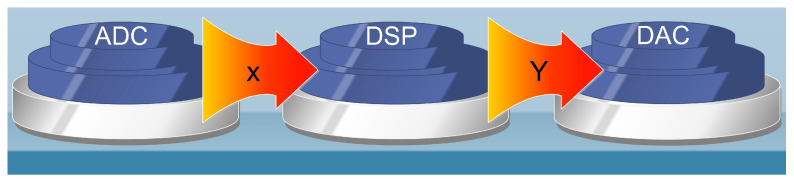
Fundamental difference equation variables.

**Figure 2:**
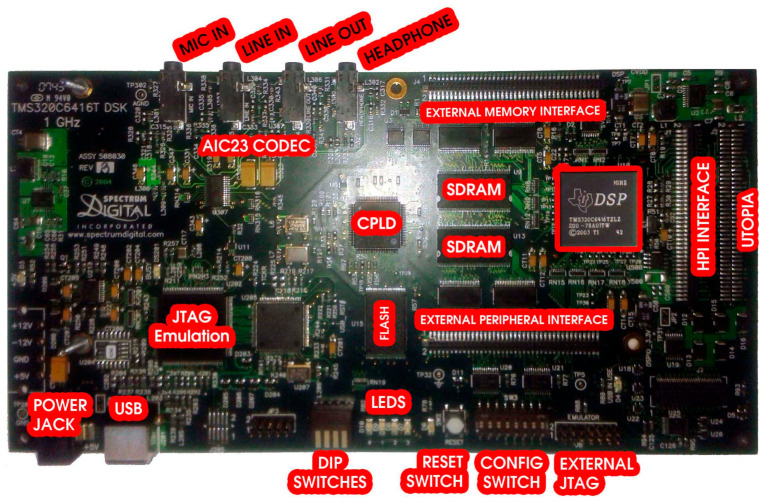
Basic DSK sectors.

It is worth mentioning that DSP algorithms are governed by Equation (1). Also, in Figure 1, the variables x and Y are graphically explained. The previous equation is called the fundamental difference equation and refers to multiply–accumulate (MAC) or sum of products (SOP). In particular, the case of MAC algorithms has been studied and developed by Texas Instruments for finding the appropriate solutions. Also, Figure 2 shows the DSK with its most important sectors. The connection to the computer is easy. Also, the declaration on a personal computer (PC) of the DSP family and operating platform (DSP starter kit—DSK) is required once, after installing MATLAB and CCS, respectively. In turn, we can connect the USB cable, the inputs–outputs of the DSP, and then its power supply. Finally, after the DSP detection by the computer, the desired code is integrated into the processor (DSP) through MATLAB and Code Composer Studio (CCS) [8].

(1) $$\Upsilon =\overset{count}{\underset{\iota =1}{\sum }}\mathrm{coeffi}\cdot x_{i}$$

## 3. DSP Implementation

In this section, the creation of signal generation systems that are pulses, sinusoidal signals, and the combined generation of the previous two by DSP [9] will be mentioned. The previous implementations helped the research progress by confirming the correct operation of the integration method by comparing the simulated signal to the corresponding measured signal. The measured signal originated from the DSP and was displayed each time on an oscilloscope. Moreover, the final implemented system was reported and discussed with the appropriate measurements from an oscilloscope and a network analyzer. Notably, the following accomplished procedure (Section 3.1) could act each time as the verification of the good theory of operation.

In particular, the code integration procedures need predefined steps to ensure no mistakes are made during implementation [5]. These could be the following in the case of working with MATLAB and CCS:

- Ensure that all cables to the power supply and to and from ADCs and DACs are attached properly while the USB cable connects the DSK board to the personal computer (PC).
- After launching MATLAB/Simulink and confirming the code correctness, TI 6000 Target (C6000 library) should be selected and inserted inside the Simulink model. Then, the target type (code generation) should be the working one of C6416DSK. For example, if the DAC is needed, the corresponding block (C6416DSK DAC) should be in the model.
- The proper arrangements should be conducted in the debug and solver categories. Afterward, the “incremental build” should be executed from the corresponding option. A sequence of code generation is that, firstly, the code is compiled in MATLAB, then CCS is initialized and in turn the assembly code will be compiled and downloaded to the target DSP. At the same time, the advancement toward DSP implementation appears in the code progression screen.
- In general, the whole procedure can take several seconds in the presence of a graphical user interface (GUI). It helps the user by making the procedure of altering some model values inside the GUI and applying the building procedure quicker, so the productivity increases as the utilized times decrease.

### 3.1. Incorporating Schematics into the DSP for Checking Proper Operation

**Figure 3:**
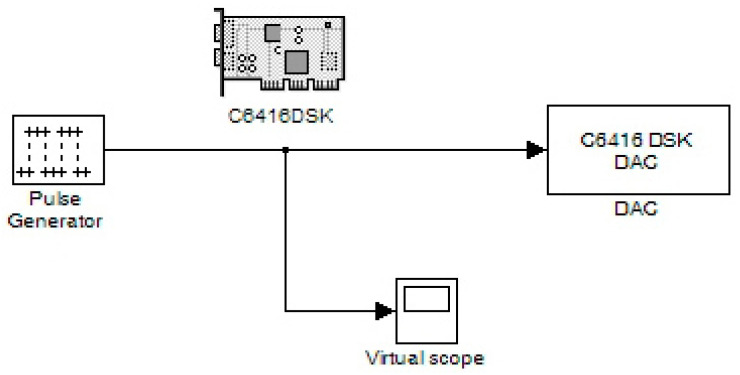
Pulse generation system.

In this sector, various digitally produced signals are shown that were created by a DSP. Specifically, Figure 3 illustrates a pulse generation system. Its characteristics are configured by the “Pulse Generator” code section. Also, three other stages include the C6416DSK, the digital-to-analog converter (DAC), and a virtual measuring instrument (scope) corresponding to an oscilloscope. The internal virtual pulse generator produced a frequency signal of 100 Hz with a sampling frequency of 48 KHz. Therefore, we had 480 points per period while the pulse width corresponded to 240 samples. This pulse width also determined the time of its high state. So, the duty cycle (pulse width) was equal to 50% of the period of the generated signal. The target code part of the DSK platform (C6416DSK) had a working frequency (CPU) of 1 GHz. The operating system was set to DSP/BIOS. It was a real-time operating system (RTOS). The board type was the model C6416DSK with a C6416 processor.

**Figure 4:**
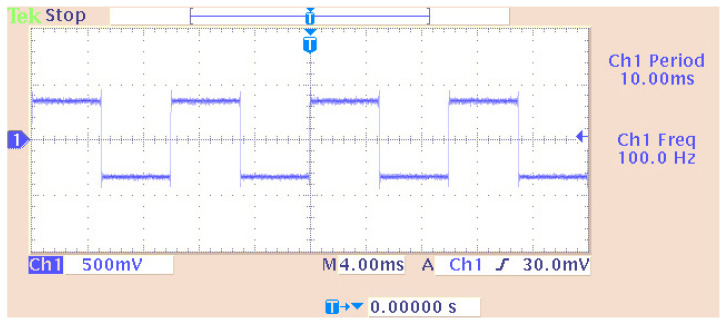
Signal (produced from a DSP) measurement on an oscilloscope.

**Figure 5:**
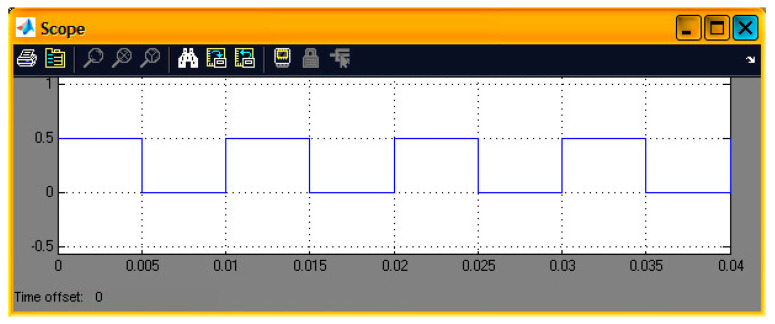
Display of a virtual signal in the time domain (scope simulation).

The DAC was set to a sampling rate of 48 KHz while the word length was the standard of 16 bits. This DAC setting helped to interpret the input word range. If this field did not exist, converting a digital signal to an analog one would have certainly produced many errors. Also, other word lengths could be 20, 24, and 32 bits which should be the same as the ADC counterparts (in the case of the use of an ADC). Also, the scaling was set to “normalize” in the value range of ±1.0. The “overflow mode” was configured to be in the “wrap” condition. In general, overflow is relative to values that are out of scale. The chosen method was considered the most efficient. Also, the virtual measuring instrument in the time domain (scope) was set to virtually measure a time range of 0.04 s. Figure 4 and Figure 5 show the output signal from the DAC (headphone) and the simulated signal, respectively. Their comparison concluded that the simulated signal was similar to the generated signal at the output of the DAC.

**Figure 6:**
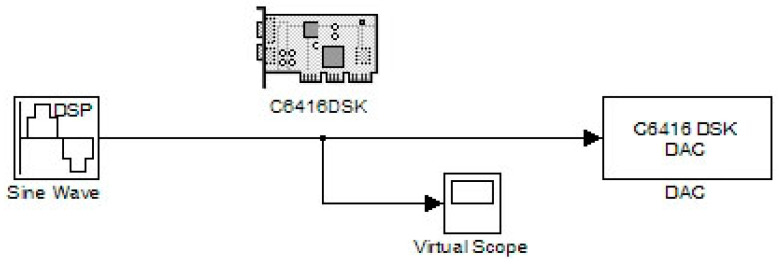
Sinusoidal generation system.

**Figure 7:**
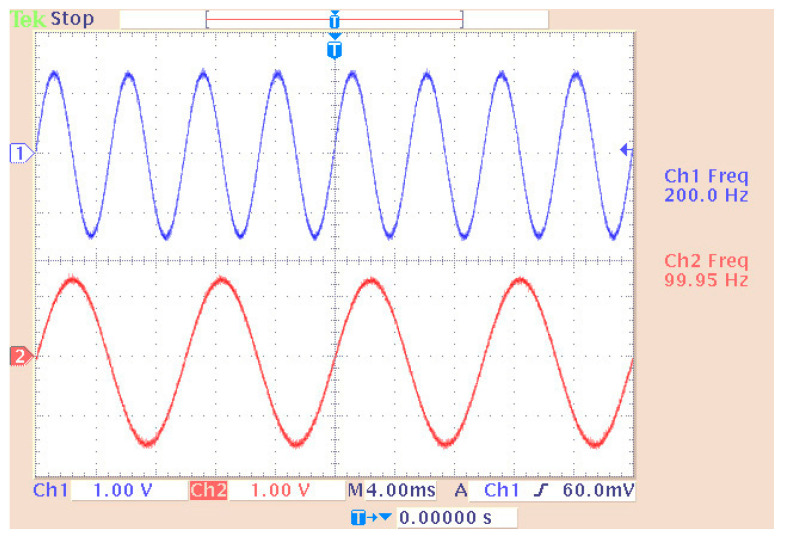
Sinusoidal plot (measurement from an oscilloscope).

**Figure 8:**
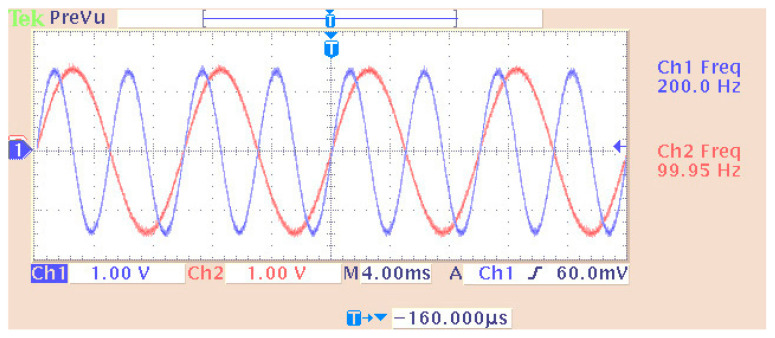
Display of overlapping channels (real-time measurement with an oscilloscope).

**Figure 9:**
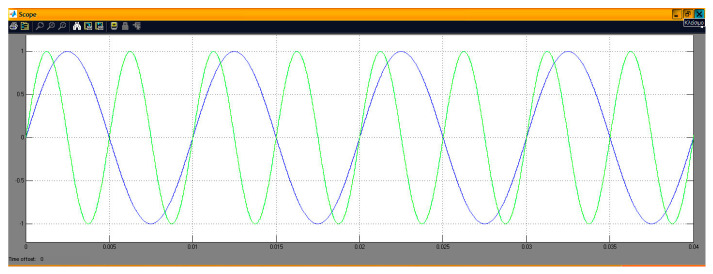
Display of overlapping channels (simulated—blue curve refers to 100 Hz while the other to 200 Hz).

Figure 6 shows a sine wave generation system with the particularity that the “Sine Wave (DSP)” stage made it possible to simultaneously generate multiple sinusoidal forms (in different channels) with independent characteristics (amplitude, phase, etc.). In this case, the desired output frequencies were 100 Hz and 200 Hz, with a sampling frequency of 48 KHz. So, the generated points per period (of the digital signal) were 480 and 240 for the previous frequencies, respectively. All other stages were the same (with similar settings) as those of the pulse generation case. The only difference lay in the overflow mode of the DAC where, in this case, the option of “saturation” was tested with complete success. The measurement (on an oscilloscope) and simulation of the two desired frequencies (100 and 200 Hz) are depicted in Figure 7, Figure 8 and Figure 9. In particular, Figure 8 shows the visual overlap of the two generated sinusoidal signals (100 and 200 Hz) on the oscilloscope screen. In this way, we can compare the simulated signals (Figure 9, where the simulation time equals 0.04 s) to those produced by the DSP (Figure 8). Finally, the comparison confirmed the agreement between simulation and practice again.

**Figure 10:**
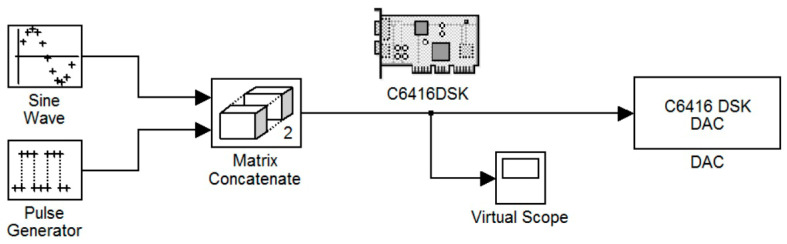
Sinusoidal and pulse generation system.

**Figure 11:**
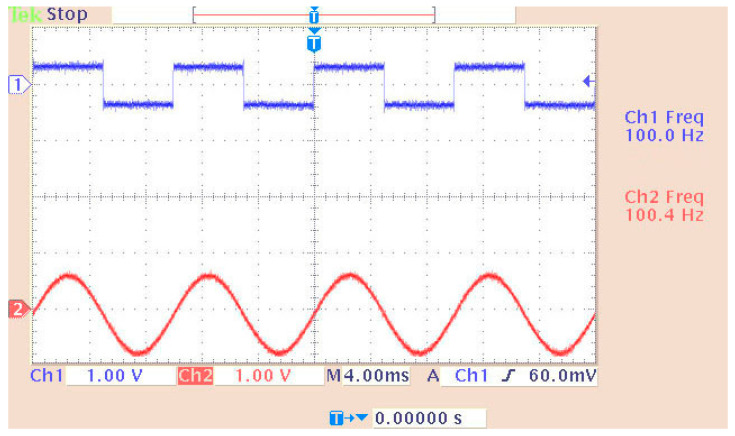
Measuring a dual generator system on an oscilloscope.

**Figure 12:**
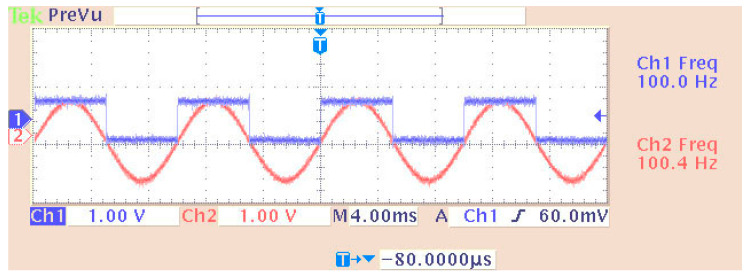
Display of overlapping channels (oscilloscope).

**Figure 13:**
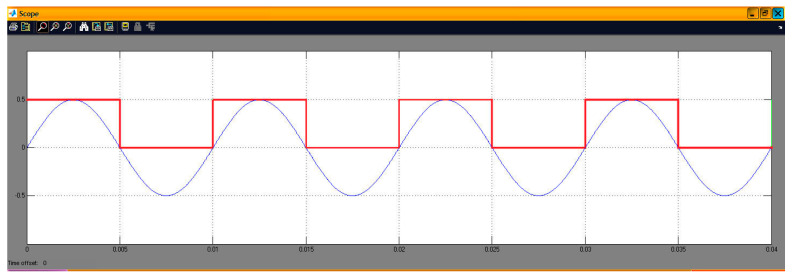
Display of overlapping channels (simulated—blue sinusoid of 100 Hz and red pulse of 100 Hz).

**Figure 14:**
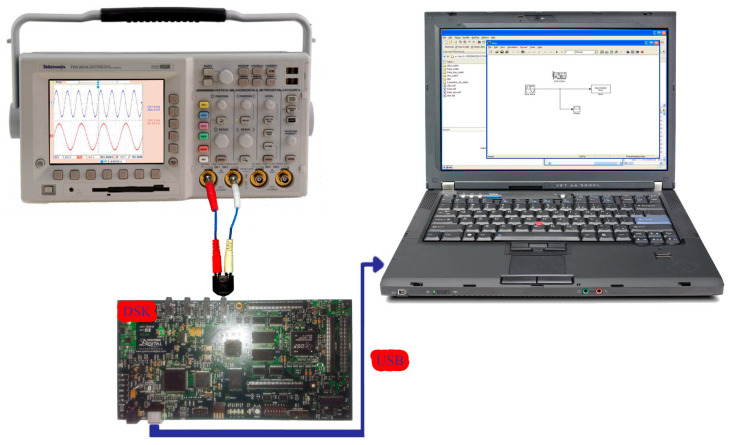
TMS320C6416T DSK connection with an oscilloscope (Tektronix TDS 3034) and computer (laptop).

Figure 10 shows the third implemented system in the DSP consisting of two virtual signal generators. These produced sine waves and pulses of 100 Hz with a sampling rate of 48 KHz. Also, in this case, the DAC operated in saturation (in case of overflow). All other settings were the same as before. A newly added option was the matrix concatenation function. This array operation concatenated the two signals. The result was the creation of two channels, each carrying one of the two generated signals. Signal measurements were conducted by using an oscilloscope (Figure 11). Also, Figure 12 shows the overlapping of the two signals to be compared visually with the simulation results shown in Figure 13. Finally, the results of the two signals’ comparison revealed the absolute agreement between simulation and measurement, certifying the correct integration method and, in general, the fine operation of the DSK system. Also, Figure 14 shows a qualitative schematic of the measurement arrangement of the system.

### 3.2. Implementation of the OFDM System in the DSP

**Figure 15:**
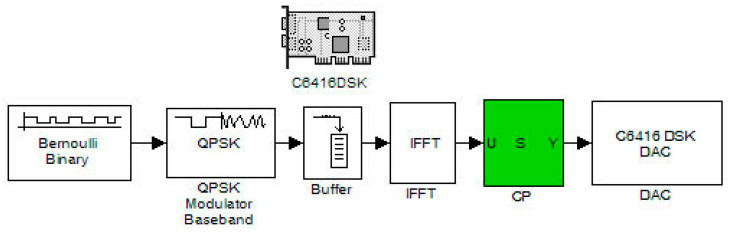
OFDM system without coding.

**Figure 16:**
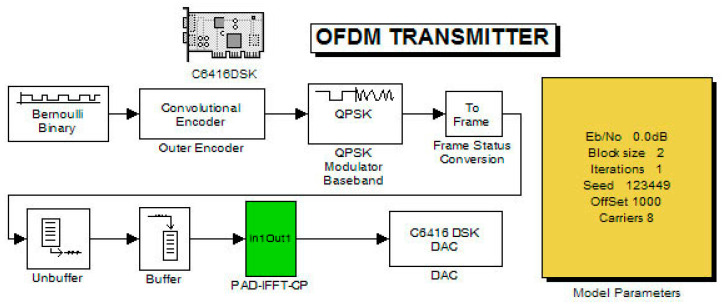
Coded OFDM system (within the green PAD-IFFT-CP layer, the additional code blocks ZP, FT, and CP are included).

**Figure 17:**
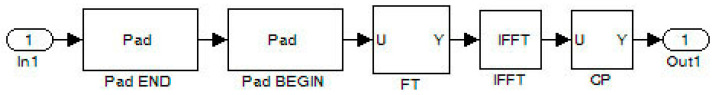
Subsystem of “PAD-IFFT-CP”, containing zero padding (ZP), frame transformation (FT), and cyclic prefix (CP).

This section consists of two stages. The first stage contains the measurements of the transmitter carried out with the help of a real-time spectrum analyzer. The second stage involves the transmitter and receiver system measurements with an oscilloscope where the transmitted pulses, after reaching the receiver, were decoded and compared with the original ones. With this technique, the total delay of the system was also easily found. Moreover, the transmitter’s case was gradually tested. Accordingly, each time, a different number of subcarriers was produced (by the DSP) for every version of the transmitter system. These versions (Systems V1 to V5) are shown below in Figure 15, Figure 16 and Figure 17. They also show the flexibility of the system toward altering the number of produced information subcarriers (known as frequency channels):

- System V1: Random binary generator with modulator and IFFT;
- System V2: Random binary generator with modulator, IFFT, and cyclic prefix;
- System V3: Random binary generator with convolutional coding, modulator, and IFFT;
- System V4: Random binary generator with convolutional coding, modulator, IFFT, and cyclic prefix (CP);
- System V5: Random binary generator with convolutional coding, modulator, addition of double zeros of at the beginning and end of the signal (double ZP), IFFT, and CP.

In the first stage, the random binary generator produced the bit “0” with a 50% probability. The convolutional encoder had a coding rate equal to 1/2, a constraint length equal to 3, and generator polynomials 7_8_ and 5_8_ with a feedback connection equal to 7. The modulator produced symbols based on a Gray constellation (00, 01, 11, 10 with corresponding phases π/4, 3π/4, 5π/4, and 7π/4) [10]. The buffering block created the parallel channels (subcarriers) and then the inverse fast Fourier transform (IFFT) was conducted. Also, when CP and ZP were used, each of them accounted for 25% of the signal.

In the case of the addition of zeros (ZP), these were transferred to the middle of the subcarriers (frame transformation—FT), as shown in [11]. For example, the OFDM subcarriers were from 0 to 2 N, and the zeros were placed in the middle of the subcarriers, i.e., in the region N. The concept of this transformation was found in the 802.11a protocol. According to Nyquist’s sampling theorem, if we have a sampling frequency of f_S_ then the maximum frequency we can detect without aliasing [12] is at most equal to f_S_/2. So, we placed the zeros in the middle of the subcarriers to accomplish subcarriers’ frequencies up to fs/2.

Also, the system models (V3–V5) contained a parameter tab (graphical user interface (GUI)) for very quick reprogramming. The tab in the GUI, in addition to the number of subcarriers, the random number generator setting (through the seed), and the size of the broadcast block, additionally contained the parameter offset (adjusting factor of the transmission speed), Eb/No, as well as the number of iterations (for future development of the system with turbo codes in practice). In detail, the GUI construction depends on Mask Editor (newer version in [13]). It refers to a user interface that includes many blocks of code in the MATLAB environment. Hence, it hides them behind an interface, while presenting only the selected predefined values by the developer. Obviously, customization exists through editing the mask. Consequently, accessing the mask editor dialog box can be performed by selecting the candidate block of code under inclusion. Then, in the model window and through the “edit” option and in turn by using the mask subsystem option, the GUI design is available. In the specific case, all the aforementioned system settings can be easily altered through the options (which can differ based on MATLAB version) relevant to “Icon & Ports”, “Icon Drawing Commands”, and “Parameters”.

**Figure 18:**
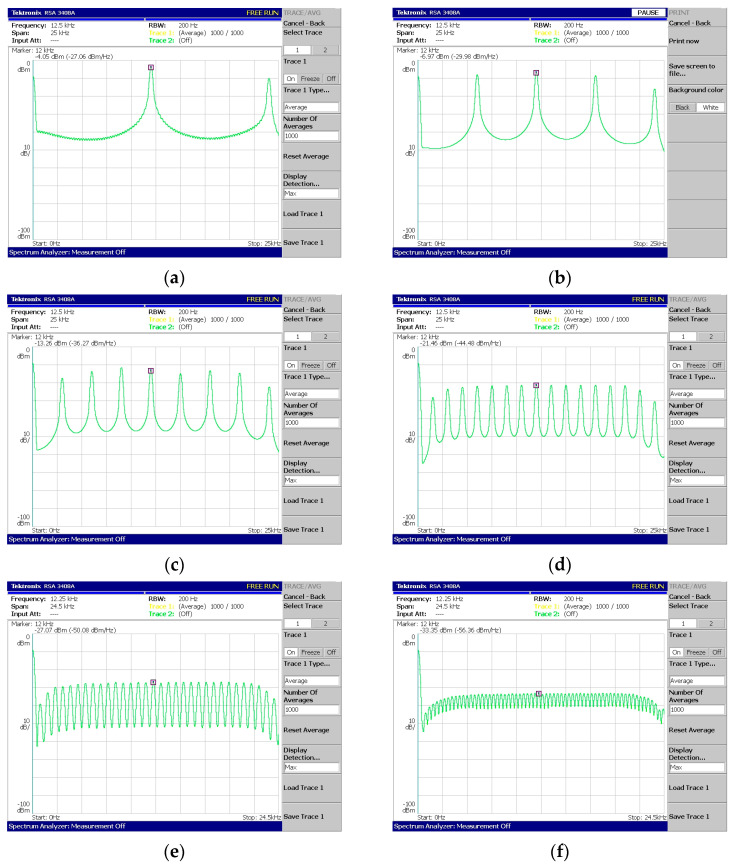
System V1—Production and measurement of (**a**) 2 subcarriers, (**b**) 4 subcarriers, (**c**) 8 subcarriers, (**d**) 16 subcarriers, (**e**) 32 subcarriers, and (**f**) 64 subcarriers.

**Figure 19:**
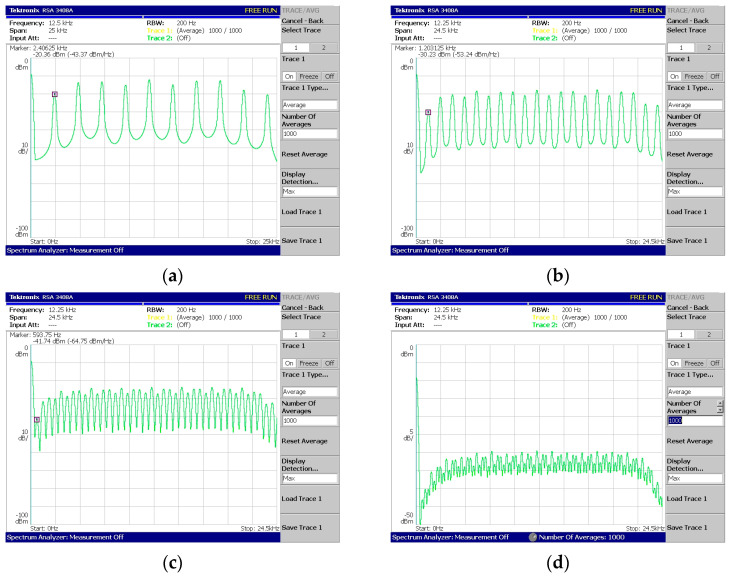
System V2—OFDM generation with CP: (**a**) 8 + 2 subcarriers, (**b**) 16 + 4 subcarriers, (**c**) 32 + 8 subcarriers, and (**d**) 64 + 16 subcarriers, where the added subcarriers are due to the cyclic prefix (CP) function.

**Figure 20:**
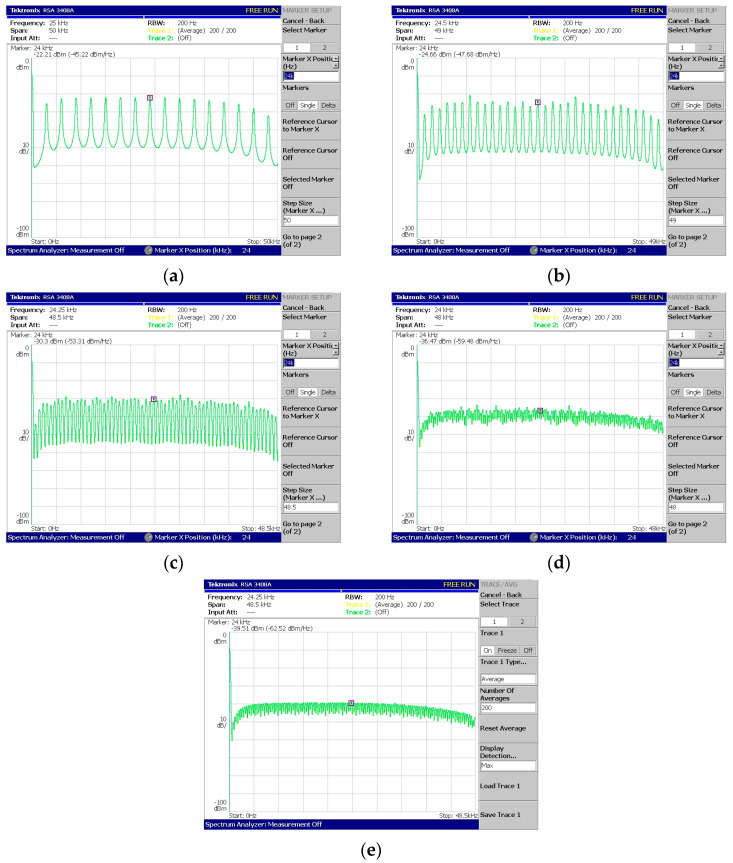
System V3—Coded-OFDM generation with (**a**) 16 subcarriers, (**b**) 32 subcarriers, (**c**) 64 subcarriers, (**d**) 128 subcarriers, and (**e**) 128 subcarriers measured using spectrum average.

**Figure 21:**
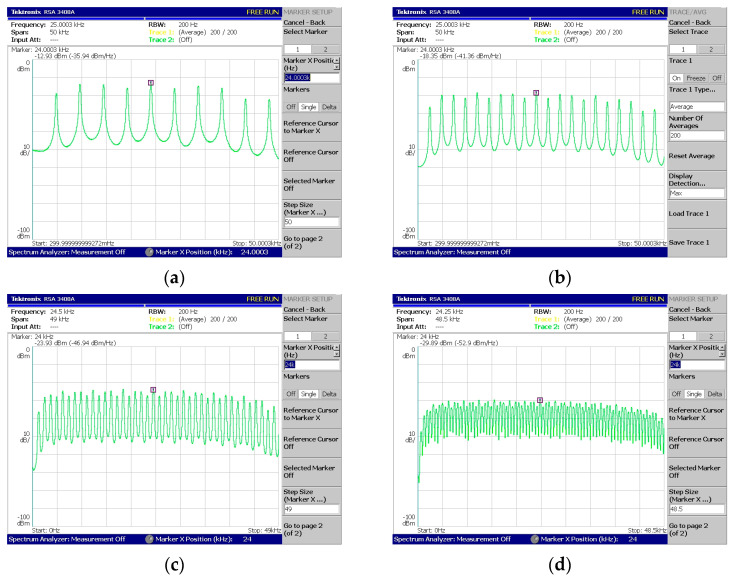
System V4—Coded-OFDM generation with CP and with (**a**) 8 + 2 subcarriers, (**b**) 16 + 4 subcarriers, (**c**) 32 + 8 subcarriers, and (**d**) 64 + 16 subcarriers.

**Figure 22:**
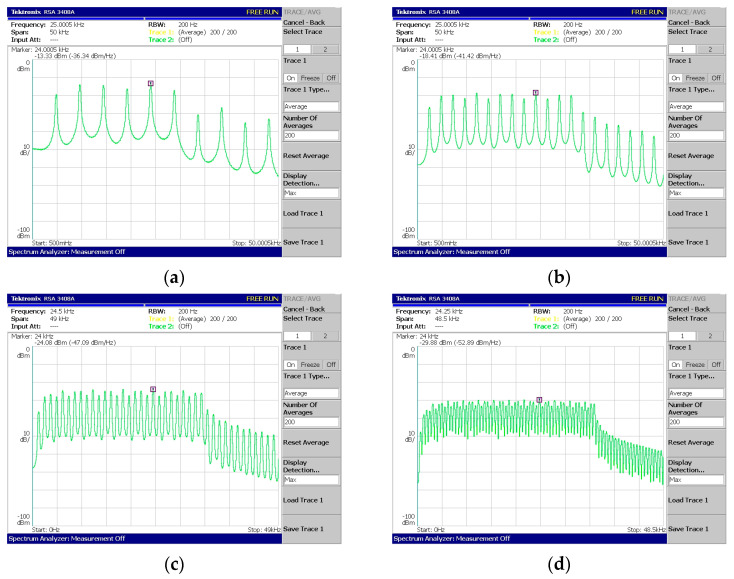
System V5—Coded-OFDM production with ZP, CP, and (**a**) 2 + 6 + 2 carriers, (**b**) 4 + 12 + 4 subcarriers, (**c**) 8 + 24 + 8 subcarriers, and (**d**) 16 + 48 + 16 carriers (X + Y + Z carriers = ZP + DATA + CP).

**Figure 23:**
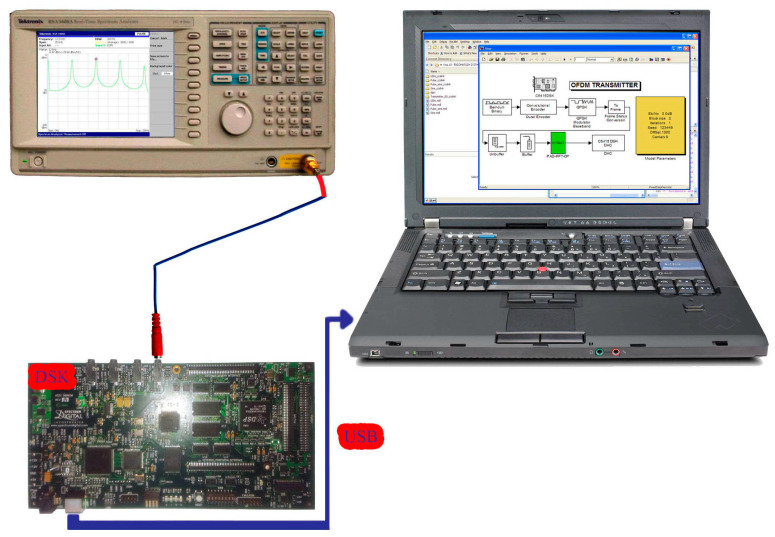
OFDM system connection with Tektronix RSA 3408A.

Regarding Figure 18, Figure 19, Figure 20, Figure 21 and Figure 22, these are acquired by measuring the system with a real-time spectrum analyzer (RSA—Tektronix RSA 3408A). Moreover, Figure 23 shows the qualitative connection between the DSK, laptop, and RSA for performing spectrum measurements.

The above measurements (Figure 18, Figure 19, Figure 20, Figure 21 and Figure 22) were acquired using the Tektronix RSA 3408A real-time spectrum analyzer while using the power spectrum averaging function. This function normalized the measured spectrum suffering from power fluctuations (PAPR). Large fluctuations in peak-to-average power have undesirable effects on system performance. If Figure 20d,e are compared, the power fluctuation of the DAC output is visible for 128 OFDM subcarriers (without using the averaging function in the spectrum analyzer). It is worth mentioning that two PAPR techniques [14,15] were successfully incorporated into the DSP but not yet measured in practice. At this stage of development, this was not deemed necessary since the goal was to integrate the transmitter and receiver system into a DSP.

In Figure 22, the phenomenon of reduced power is visible for the subcarriers from a specific frequency to the highest-produced one. It is due to a percentage of the subcarriers corresponding to ZP and CP. For example, in Figure 22c, the number of produced subcarriers is equal to forty of which eight are ZP and eight are CP. But, as the last samples of the signal were copied to the beginning (CP procedure), these contained (within the eight copied samples) the four zeros coming from the ZP. Therefore, the twelve highest frequencies (CP + ZP) were expected to be attenuated (from 34.8 KHz up to 48 KHz).

**Figure 24:**
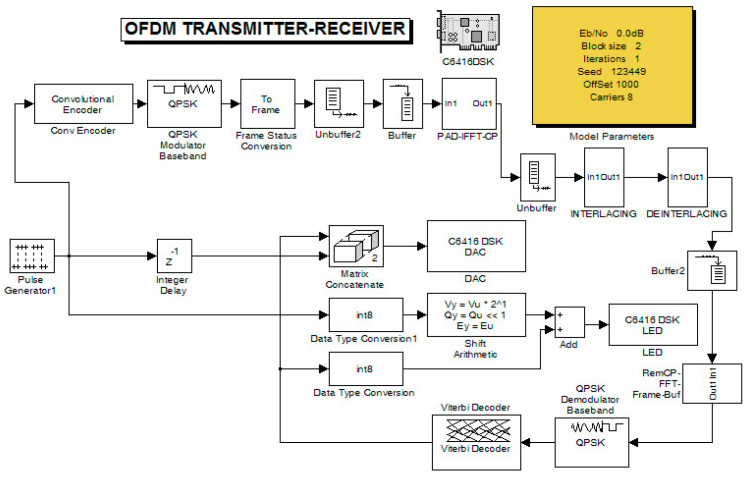
OFDM transmitter and receiver.

The second stage of measurements was conducted on the final version of the embedded platform (on the DSP). The primary measurements on the oscilloscope concerned the total delay of the system without the presence of noise. Therefore, the OFDM transmitter and receiver integration into the same DSP was performed using an ADC and DAC between them. The transmitter–receiver system (Figure 24) consisted of a pulse generator that periodically produced “1 s” and “0 s”. These were convolutionally encoded while the resulting signal was QPSK modulated.

**Figure 25:**
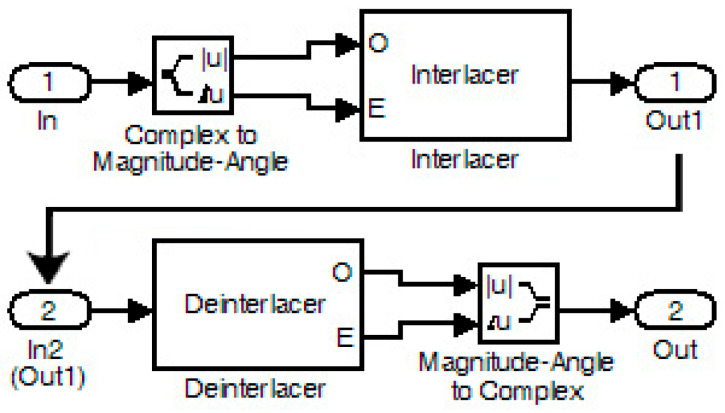
Stages of interlacing (In→Out1) and deinterlacing (In2→Out).

**Figure 26:**
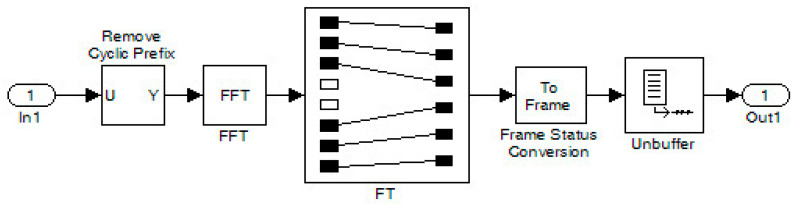
Stage of RemCP–FFT–Frame–Buf.

In coded QPSK, zero padding (ZP) and frame transformation (FT) were applied, and afterward, with the IFFT function, the final coded OFDM was produced. Also, a cyclic prefix (CP) was added to this signal. The resulting signal had to undergo unbuffering (frame-to-frame conversion into one-dimensional samples) to pass through the channel. Although this procedure was not necessary, it was included to prepare the model for a future measurement step. The signal was then split into amplitude and phase angle components and combined using the interlacing technique. In the receiver, deinterlacing was carried out where the separated signal amplitude and phase angle components were combined and sent to the following stages of the receiver. This is depicted in Figure 25. Also, other stages of the receiver contained the inverse operations of the transmitter for recovering the signal. In particular, the procedures for removing zeros (ZP) and cyclic prefix (CP) are shown in Figure 26. After the Viterbi decoder, the decoded signal was fed along with the original signal (from an internal generator) to the DAC and then to the oscilloscope.

**Figure 27:**
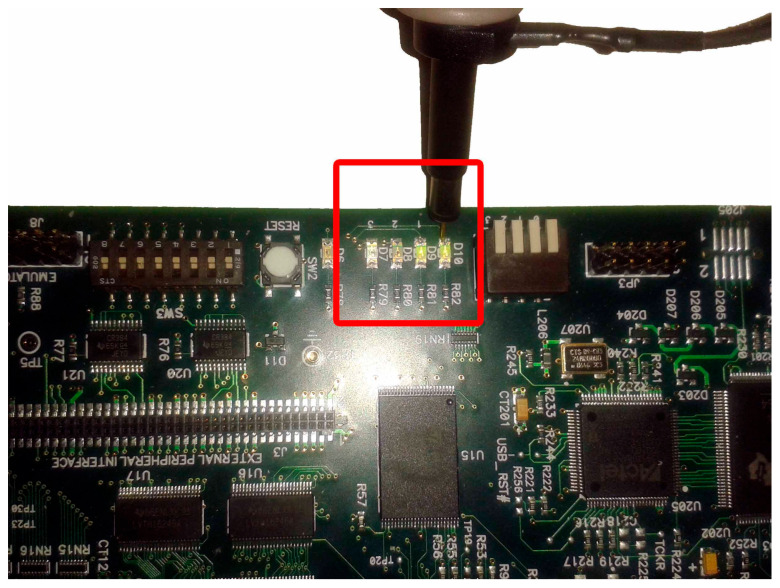
LED signal measurement with oscilloscope’s probe.

**Figure 28:**
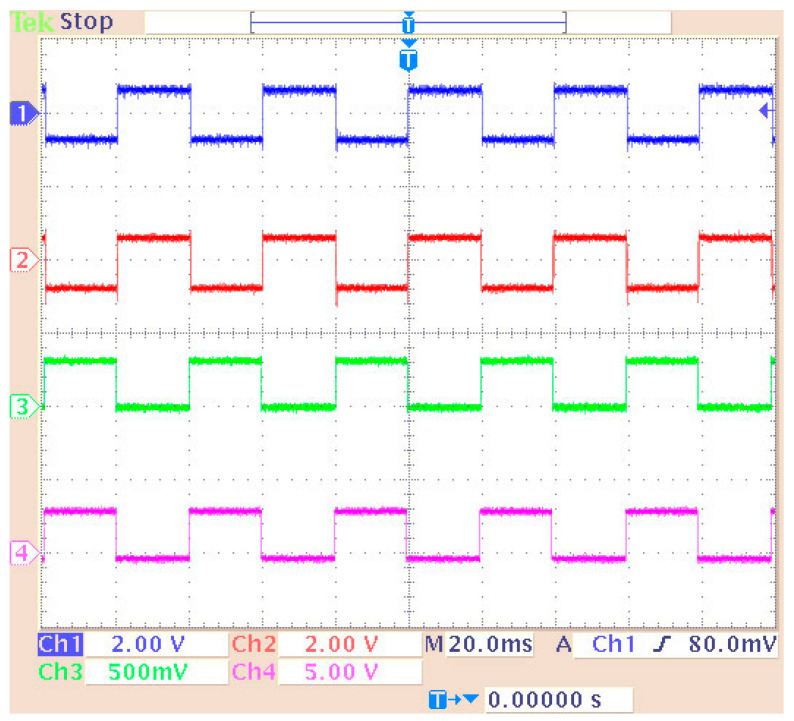
Measuring signals using an oscilloscope: The output of the DAC is shown on Channel 1 (generator data) and Channel 2 (Viterbi decoder output signal). The measurement of the LEDs is shown on Channel 3 (generator data—LED1) and Channel 4 (Viterbi decoder output signal—LED0).

The original signal of the internal generator and the received signal (decoder output) were output to the LEDs of the DSK (Figure 27) while they were measured using an oscilloscope probe. In this phase of the measurements, the DAC and ADC of the DSP were used as the output of the transmitter and receiver input, respectively. Therefore, an alternative way was utilized for measuring the signals (at the LEDs’ terminals) since the DAC and ADC could not be used for measurement purposes (because their outputs were driven to an oscilloscope). In this case, other parts of the code had to be integrated into the DSP for transferring the signals to the LEDs. A segment of the code (data type conversion—Figure 24) converted the produced and received signal into 4 bits (1 bit for each LED and hence the lighting of the four LEDs represented integers from 0 to 15). Also, a numerical shift (shift left arithmetic) was included so that each signal fed a different LED. Therefore, the originally generated signal blinked LED1 (1st bit—2^0^) while the signal from the receiver blinked LED0 (2nd bit—2^1^). Also, the LEDs in the low operating state were lit. So, every low level corresponded to bit “1”, while every high level corresponded to bit “0”. The measurement method is shown in Figure 27. Also, the delay time of the transmitter–receiver system was calculated as 1 ms by comparing the transmitted signal with the received signal on the oscilloscope. Figure 28 shows that the delay was factored in and therefore the signals from the generator coincided with those from the receiver. To achieve this, the originally generated generator signal had to be delayed by 1 ms. The built integer delay stage (in the DSP) was set with a sample time equal to 0.001 s (i.e., 1 ms) and the “number of delays” equal to one (i.e., the delay is applied only once). All other settings of the code segments were similar to those of the previous measurements. The measurement wiring was similar to that of Figure 14 while the achieved measurements from an oscilloscope are shown in Figure 28.

**Figure 29:**
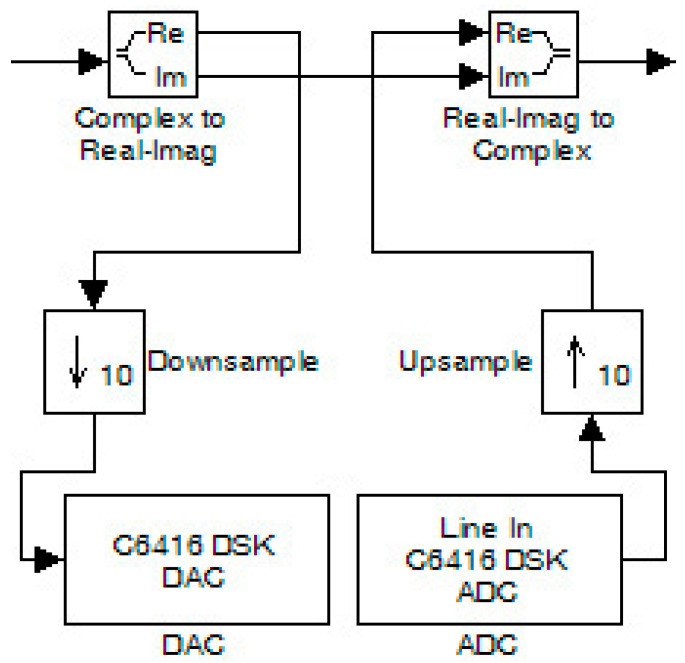
Modification of OFDM system for measuring in the presence of ADC and DAC.

The OFDM system was then measured in the presence of a DAC (at the transmitter output) and ADC (at the receiver input). The system modification involved changing the “interlacing” and “deinterlacing” code sections with new wiring at this circuit location. This wiring appears in Figure 29. There, the signal was split into two components. The imaginary part was driven directly to the receiver for synchronization purposes, while the real part of the signal went through the ADC and DAC. As the two converters had to work with the same data rate, downsampling was performed by removing samples, while the reverse process (upsampling) was performed at the receiver to increase the samples. Also, the ADC and DAC were configured with a 16-bit word size, 8 KHz sampling rate, normalized scaling, and overflow mode set to saturation.

**Figure 30:**
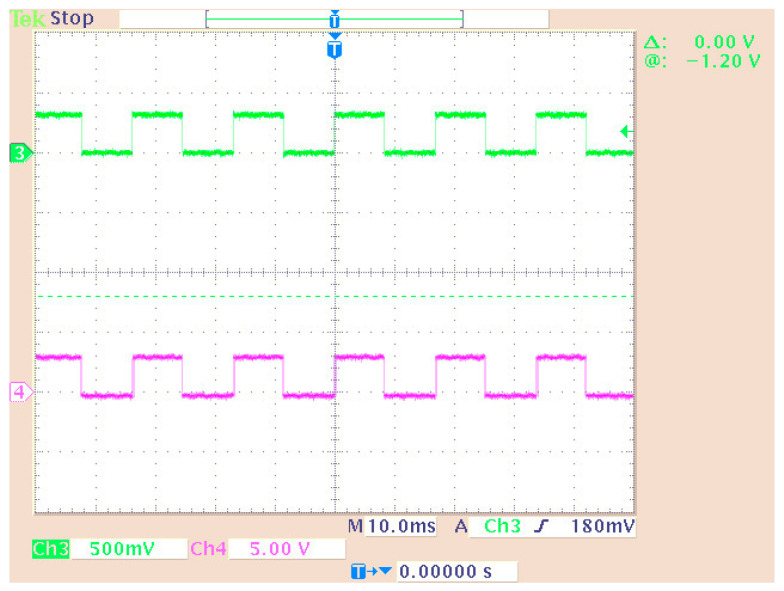
Measurement on system LEDs (Channel 3—initial signal on LED0 and Channel 4—final signal on LED1).

**Figure 31:**
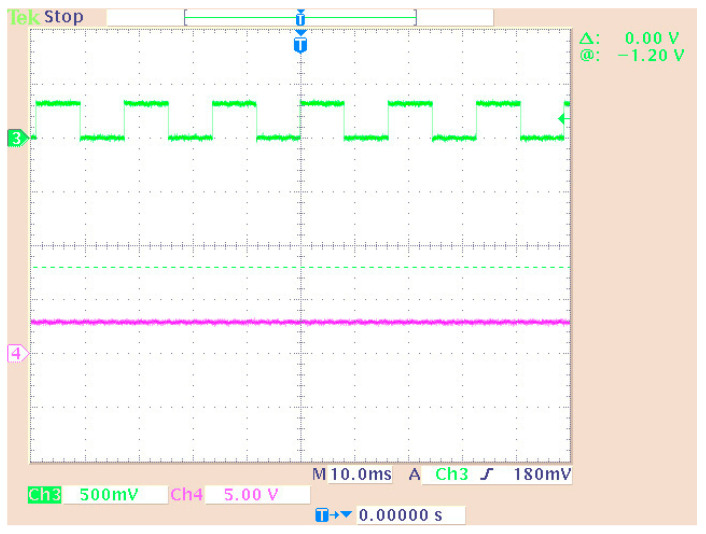
System measurement without imaginary part connection (Channel 3—initial signal on LED0 and Channel 4—final signal on LED1).

**Figure 32:**
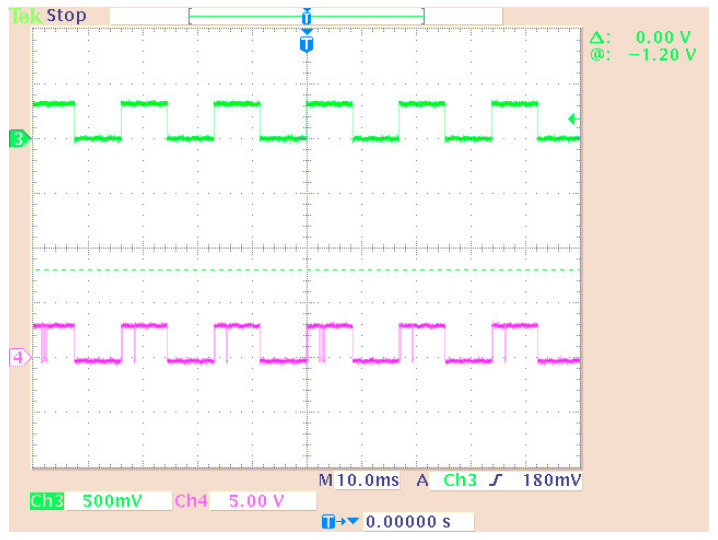
System measurement without upsampling (Channel 3—initial signal on LED0 and Channel 4—final signal on LED1).

The initial (from the generator) and final (decoder output) signals were compared for the cases with (Figure 30) and without (Figure 31) connecting the imaginary part of the signal to the receiver. Also, the case where only “upsampling” was missing from the OFDM scheme was measured (Figure 32). Then, the system showed problems in fully recovering the signal. Specifically, when the signal’s imaginary part was missing in the receiver, the signal recovery was not feasible.

## 4. Proposal of Potential System Integrations inside Modern Warfare Environment

In this section, a brief suggestion concerns a proposal of integrating the OFDM system inside systems such as an unmanned aerial vehicle (UAV) for modern operations [16]. As already known, apart from civil operations (e.g., usage of UAVs) that need real-time pattern recognition [17], a variety of warfare schemes could benefit from multiple orthogonal carriers and a spectrum upon request. It could provide the ability to change the transmitting and receiving properties of a system at any time based on the requirements of the scenario and its conditions. Consequently, the following proposals could offer better resistance to channel interception or/and antidrone techniques:

1. The initial check of the system should contain simple procedures to ensure the proper operation based on Section 3.1 relevant to the implementation of simple schematics/code on the targeted platform.
2. A flexible system (e.g., UAV) could quickly change the utilized frequencies (e.g., GUI in Figure 16) automatically while it would be more immune to channel variations, attenuation, and signal interception attempts.
3. The quick alteration of the produced spectrum utilizing only some of the encrypted orthogonal carriers for safety reasons (to not be intercepted) and for spectrum economy based on an artificial intelligence (AI) entity could furthermore enhance the overall performance.
4. On the other hand, radars incorporating the smart strategy of using AI techniques while having the capability of working on a vast spectrum and orthogonal carriers [18] could better contribute to seamless detection.

**Figure 33:**
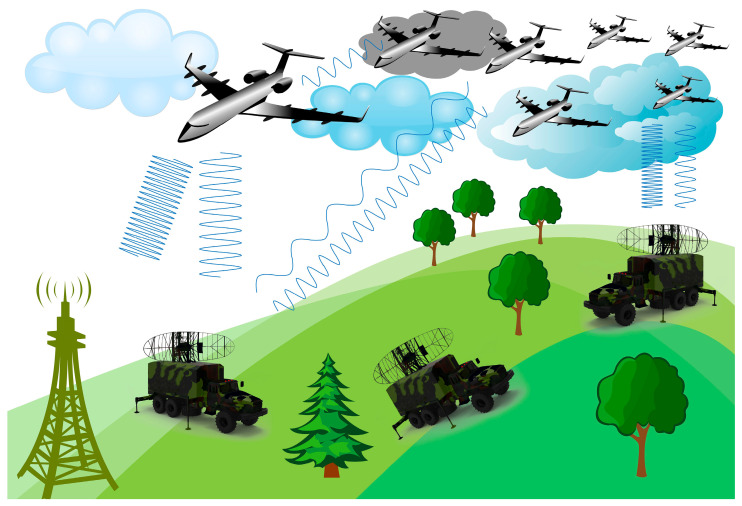
A qualitative visualization of a modern warfare scenario.

As mentioned, the third point was to qualitatively show some paradigms of applying such a system inside a modern warfare environment and how it could respond. Notably, some of the illustrated proposals in Figure 33 concern radars versus UAVs that are struggling to prevail over each other with the usage of various subcarriers. Specifically, in Figure 33, a UAV (with the role of a tracker) tries to interfere with a radar system in conjunction with other ones while the rest could be the silent-killer teammates. Consequently, in the case of the UAV trackers being detected at some time by the remaining radars, the other UAVs that are not detected will have caused great damage to the enemy infrastructure.

**Figure 34:**
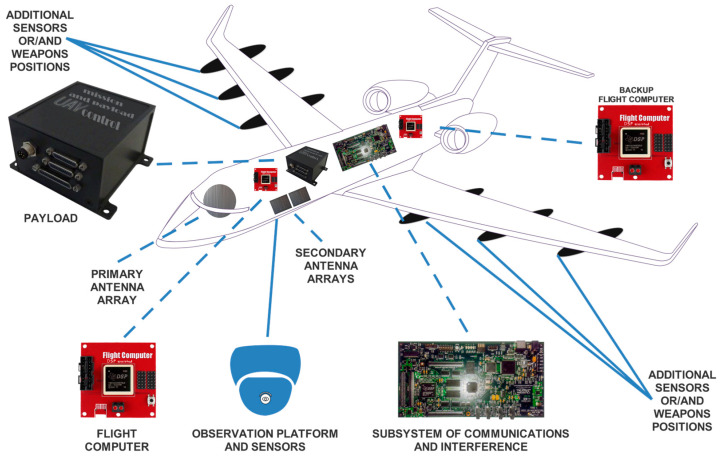
Typical structure of an advanced UAV system.

To further analyze a part of this warfare scenario, let us introduce a UAV that incorporates the proposed technology of OFDM along with a typical architecture of UAVs [19] but is enhanced with additional antenna arrays and a backup flight computer system. The main points of interest (in such a UAV system) are reported along with its operating principle. A graphical depiction of breaking down the system into its basic subsystems appears in Figure 34.

In particular, a UAV system specially designated for warfare scenarios tends to range from a relevant simple aircraft that conducts automated operations with several sensors to a more complicated system that detects, recognizes, processes, and attacks, always depending on the goal that it has to accomplish. As far as its airframe, this should meet all the edge technology requirements relevant to new materials and enhanced durability, and its size depends on the planned application. Also, the overall system has to process a vast amount of information ranging from detecting and recognizing the scanned area to conducting complex mapping for recognizing the potential enemy structures, facilities, and personnel. Some typical sensors worth mentioning are cameras, GPS, accelerometers, pressure sensors, etc. Specifically, a typical but enhanced UAV system (in a warfare scenario) could have the following structure:

1. The airframe of the UAV should consist of composite materials [20], thus being lightweight and contributing to low observability with a modular frame if possible and giving the capability of conducting various missions that require low or high overall load.
2. The payload with its controller (which can be a DSP) includes all the needed sensors for every mission while sustaining a modular strategy. Some ordinary detectors are high-analysis cameras, thermal, pressure, and humidity sensors, etc. All the obtained information can lead to a fusion for better processing of the most meaningful data and transmitting them back to the base/remote station.
3. The flight computer is the most crucial part of a UAV as with the help of its corresponding sensors such as GPS, gyros, accelerometers, etc. the system flies within the framework of approved instructions (originated by the base or a remote station). As this signal processing unit is vital, a similar one named “backup flight computer” can be implemented in a different part of the airframe for safety reasons. In this way, if the primary flight computer fails or is hit by an enemy unit, then the backup system will provide at least adequate instructions to the system for bringing it back to its base or making it a killer drone.
4. The communications subsystem can include, except the antenna arrays, an advanced DSP platform that can sustain satcomm and microwave links. Implementing the OFDM technique could compensate for signal attenuations and complete loss of radio channels due to a noisy environment. For example, considering an OFDM telecommunication system utilizing more than 1024 subcarriers, it is clear that if some are lost even from intended interference, the remaining ones will still sustain high-quality communications for transmitting real-time high-analysis video. Furthermore, having more than one DSP with the appropriate antennas, intended interference could be produced in various frequency bands depending on the transmitting–receiving band limits. Nevertheless, these limits can be altered as these kinds of systems (DSP platforms) are modular with enhanced upconverters for offering seamless performance.
5. Antenna arrays [21] can also follow a modular strategy for being changed if their technology is surpassed. Accordingly, secondary integrated antenna arrays can offer better performance and give the capability not only to act supplementarily but to instantly perform another task (e.g., transmitting electromagnetic interference—EMI) against enemy-targeted systems.
6. Finally, there should always be provision for additional sensors or weapon slots. Moreover, these are accounted for in the payload summary. Specifically, new kinds of sensors could be mounted under the wings for specific missions with the required missiles or attack systems. Furthermore, relevant to weaponry, the system should have the capability of being updated wirelessly. Imagine having advanced DSPs inside the missiles’ electronic platforms that are updated shortly with the proper algorithm for raising the efficiency of the relevant weapon or sensor [22].

## 5. Comparative Study

The proposed OFDM system, to the author’s knowledge, has not been implemented in the TMS320C6416Τ DSP Starter Kit (DSK) and measured in this specific methodological context elsewhere. Hence, to find similar works and in the context of similar technology to the aforementioned DSK, the research terms “OFDM” and “C6416” were used in Google Scholar for the time range of the last decade (2015–2024) (searching performed on 21 March 2024 twice to avoid omitting works). The procedure returned twenty-two (22) works. These included papers, course structures, theses, or non-relevant documents. Nevertheless, they were examined and it was found that: (1) three were relevant to specific industry standards but did not examine new types of OFDM nor flexible techniques of implementing or measuring, (2) one was based on MIMO-OFDM without adding new and specific contributions to OFDM, (3) another one was relevant to massive MIMO, (4) one was about algorithmic production relevant to OFDM systems, (5) eight documents were about course structures/syllabus, (6) two were the same documents as the others, and finally (7) one work was relevant to filtering and one was a review. Hence, the remaining works for comparison were four.

**Figure 35:**
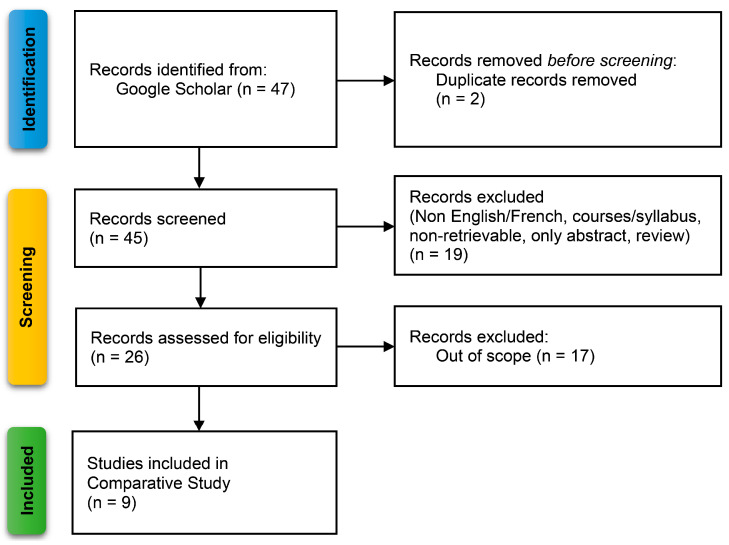
Flow diagram of procedures selecting the documents for comparison.

To find more comparable works, search terms were “relaxed” to include a type of architecture similar to TMS320C6416 relevant to TMS320C6713 DSK. For better results, the search terms were “OFDM + (C6416 OR C6713)”. All other restrictions were kept the same, concluding with forty-seven (47) results for the period of 2015–2024. Similarly, excluding non-English/French documents, out-of-scope works, courses/syllabus, non-retrievable documents, only abstracts, reviews, and duplicate files, the search produced nine documents. The selection procedure appears in Figure 35 (where “meas.” stands for measurements and “extsv” for extensive).

The incorporated works in the comparative study include various implementations such as in TMS320C6713, WARP test-bed, multicore board, TMS320C6416Τ, etc. Also, some tend to focus on emulation testing while there is a definite lack of correlating them with warfare scenarios or advanced multisensory applications. Nevertheless, the studies reported in Table 1 include unique features and extensive work. Hence, Table 1 contrasts all the works if possible, plus featuring each in brief (highlights).

## 6. Restrictions

In this paper, an OFDM platform has been presented along with its implementation in a DSP. Some explanations and restrictions are intended to clarify some crucial aspects of this work:

1. The OFDM system output spectrum is limited due to DAC restrictions that could exhibit output frequencies up to 96 KHz. This frequency range is low but the main idea was to exhibit the technique of easily implementing such a system in a well-known DSP.
2. Concerning the goal of implementing these kinds of DSP platforms (e.g., TMS320C6416Τ) in a high-frequency system, it is possible on the condition of adding other components. For example, if there is a demand for the signal to reach nearly 900 MHz [32], then there is a necessity to add a new module on the DSK board that would be an additional DAC or even an field programmable gate array (FPGA) capable of producing higher output frequencies. It could output signals to a low intermediate frequency (IF) (e.g., 10–20 MHz) to produce a well-generated signal (minimal distortions) and then drive it to an ultra-high-frequency (UHF) front-end. The latter could be a mix of filters and local oscillators providing a final signal upconversion within the targeted frequency range [32]. Compatible technology applies to the receiver’s part for reverting the signal to its original frequency (ADC input in the receiver’s DSP system).
3. Similar to the previous need for working in higher frequencies, and being aware of new emerging applications demanding even higher frequencies, a solution could be based on microwave design, e.g., modular tactics such as upconverting the signal beginning from the DSP generator to the frequency limit where distortion is minimal and then following this procedure until the generated frequency reaches the desired value.
4. Regarding various techniques involving either channel equalization or estimation, these will probably be implemented as soon as the system is ready based on the aforementioned techniques for wireless operation. Also, a new system version will be examined only with advanced turbo coding as the utilization of forward-error correction (FEC) exhibits adequate results by itself [11].
5. The warfare scenario is purely qualitative as it simply introduces the potential applications of such an OFDM platform to modern tactics with the presentation of a potential UAV system working with the proposed technology.
6. Potential add-ons of this work could be the implementation of such an OFDM platform to a more sophisticated system using AI [33] that could sustain centimeter or millimeter technologies working on a big-data basis.

## 7. Conclusions

In this paper, an advanced integration of an OFDM system is presented. Firstly, simple code was integrated, confirming the correctness of the implementation technique on the DSK platform. Subsequently and after the OFDM’s integration, the system’s measurements in the time and frequency domain again confirmed its correct operation. The measurements in the spectrum analyzer confirmed the proper system’s response in the frequency domain as expected from theory and simulations in each case. Additionally, with the use of an oscilloscope, the total delay of the system was found, which was used to confirm the good theory of operation of the integrated transmitter–receiver system. This was achieved by sending pulses that were fully recovered as shown by their comparison with the ones originally produced (on an oscilloscope). Also, some potential applications are presented briefly regarding some warfare scenarios using UAVs that could include enhancements such as fault diagnosis and tolerant control [34].

Future improvements of the system include incorporating PAPR reduction techniques into it. Specifically, PAPR can cause fluctuations in the output signal power and, in turn, this condition provokes a chain reaction of events apart from increasing the overall cost. These include problems especially with DACs operating in an extensive dynamic range and power amplifiers that need to work in larger linear regions to avoid spectral growth and probably some out-of-band noise [14]. Regarding incorporating PAPR reduction in a DSP, it will involve testing various techniques (integrated code in a DSP) at the implementation level to find the most appropriate ones. It will be accomplished by measuring the fluctuation of power levels after the DAC outputs of the DSP system. Then, the type of coding will be upgraded with iterative decoding techniques in practice [11,35]. Relevant to this type of coding technique, it is commonly reported in the literature that their efficiency ranges from the superiority of bit-error-rate (BER) performance to power efficiency. For example, the BER with the utilization of only two decoding iterations, in a parallel concatenated convolutional coding (PCCC) scheme and for an OFDM of 2048 carriers, can be below 10^−5^ in a noisy environment (additive white Gaussian noise (AWGN)), while the convolutional coding technique exhibited a value slightly larger than 10^−3^ [11]. Consequently, the system with a low BER needs no retransmission, leading to lower power consumption. The implementation will involve, apart from integrating the relevant code in two DSP systems, testing under noisy conditions (using wired and wireless communication channels) to retrieve their ability to withstand such noise in terms of BER.

## Figures and Tables

**Table 1:** Comparison between the implemented system and related works.

Ref.	Experimental Setup	Real-Time Frequency Meas.	Real-Time Time-Domain Meas.	ZP and CP Meas.	System Delay Meas. or Other Delays	GUI	Highlights/ Warfare Scenario
[23]	Loaded code in TMS320C6713	No (simulated)	No	No	No	No	RS coding in TMS320C6713 /No warfare
[24]	Loaded code in TMS320C6713	No (simulated)	No	No	No	No	Verifying SISO-OFDM code in DSP /No warfare
[25]	ANSI C code generation intended for TMS320C6713	No (simulated)	No (simulated)	No	No	No	Easy OFDM design /No warfare
[26]	Implemented code in TMS320C6678	No	No	No	No	No	Recovered image after fading channel emulation /No warfare
[27]	Implemented code in WARP test-bed	Yes	No	No	No	No	Wireless pilots using two dual band antennas /No warfare
[28]	Implemented code in a multicore board	No	Yes	No	No	No	Reconfigurable platform for underwater IoT /No warfare
[29]	Implemented code in TMS320C6713 and Xilinx FPGA-Virtex5	No	No	No	Yes	No	Estimating computation times /No warfare
[30]	Utilization of acquisition card	Yes (emulated)	Yes (emulated)	No	No	Yes	Designing hardware in the loop underwater comm. emulation /No warfare
[31]	Implemented code in TMS320C6713	Yes (not extsv)	Yes (not extsv)	No	Yes	Yes	Underwater acoustic implementation /No warfare
This work	Implemented code in TMS320C6416Τ	Yes (extsv)	Yes (extsv)	Yes (extsv)	Yes	Yes	OFDM scenarios and measurements /Warfare

## Data Availability

Data are available upon reasonable request.
